# Superb microvascular imaging in guiding targeted biopsy of prostate cancer: A protocol for systematic review and meta analysis

**DOI:** 10.1097/MD.0000000000023604

**Published:** 2020-12-18

**Authors:** Cong Wang, Ye Tao

**Affiliations:** Ultrasound Department of the First Affiliated Hospital of Dalian Medical University, Dalian City, Liaoning Province, China.

**Keywords:** meta-analysis, prostate cancer, superb microvascular imaging

## Abstract

**Background:**

Studies suggested superb microvascular imaging technology to guide prostate cancer biopsy could improve the positive rate of draw materials. The present meta-analysis aimed at determining the accuracy of SMI in the location diagnosis for prostate cancer.

**Methods:**

We will search PubMed, Web of Science, Cochrane Library, and Chinese biomedical databases from their inceptions to the October 31st, 2020. Two authors will independently carry out searching literature records, scanning titles and abstracts, full texts, collecting data, and assessing risk of bias. Review Manager 5.2 and Stata14.0 software will be used for data analysis.

**Results:**

This systematic review will determine the accuracy of superb microvascular imaging in guiding targeted biopsy of prostate cancer.

**Conclusion:**

Its findings will provide helpful evidence for the accuracy of superb microvascular imaging in guiding targeted biopsy of prostate cancer.

**Systematic review registration:**

INPLASY2020100117.

## 1 Introduction

Prostate cancer is one of the most common malignant tumors of male urinary system, which derives from the prostate epithelium.^[1]^ Prostate cancer has a relatively good prognosis, and even has the possibility of cure, so early diagnosis is of great significance.^[2]^ Prostate specific antigen is helpful in the detection of prostate cancer, but the effect is not significant.^[3]^ Transrectal ultrasound guided biopsy of prostate nodules is the main way to detect prostate cancer.^[4]^ However, it is difficult to show prostate cancer by conventional transrectal ultrasound because of its small size or atypical echo, which leads to missed diagnosis of puncture.^[5]^ Prostate cancer has many neovascularization and abundant blood supply, but the traditional color Doppler flow imaging can not show the blood flow of cancer very well.^[6]^ As a novel ultrasound technique, superb microvascular imaging (SMI) can quickly, simply, and noninvasively study the microvascular distribution in the tumor and evaluate the microvascular perfusion.^[7]^ It can be used to guide the localization of suspicious areas of prostate cancer negative by conventional transrectal ultrasound, which is helpful to improve the puncture positive rate of malignant lesions.^[8]^ Studies suggested SMI technology to guide prostate cancer biopsy could improve the positive rate of draw materials.^[9,10]^ However, there is no systematic review or meta-analysis providing evidence to determine whether SMI is an ideal method to guide prostate cancer biopsy. Therefore, the present meta-analysis aimed at determining the accuracy of SMI in the location diagnosis for prostate cancer.

## 2 Materials and methods

This study will be conducted in accordance with the PRISMA (Preferred Reporting Items for Systematic Reviews and MetaAnalyses) guidelines and the protocol has been registered in the INPLASY (INPLASY2020100117).

### 2.1 Eligibility criteria

#### 2.1.1 Type of study

This study will only include high quality clinical cohort or case control studies.

#### 2.1.2 Type of patients

The patients should be those who undergone prostate biopsy. We will not apply any restrictions of race, age, education background, and economic status.

#### 2.1.3 Intervention and comparison

This study compare SMI with pathology for diagnosing prostate cancer.

#### 2.1.4 Type of outcomes

The primary outcomes include sensitivity, specificity, positive and negative likelihood ratio, diagnostic odds ratio, and the area under the curve of the summary receiver operating characteristic.

## 3 Search strategy

PubMed, Web of Science, Cochrane Library, and Chinese biomedical databases will be searched from their inceptions to the October 31st, 2020. We will not impose any limitations to language and publication status. The search strategy will be built with the assistance of a professional librarian. The search strategy for PubMed is shown in Table 1. Other online databases will be used in the same strategy.

**Table 1:** Search strategy sample of PubMed.

Number	Search terms
1	Prostate tumor or prostate cancer or prostate neoplasm
2	Biopsy or puncture
3	Superb microvascular imaging or superb micro-vascular imaging or SMI
4	and 1–3

### 3.1 Data extraction and quality assessment

Two authors will independently select the trials according to the inclusion criteria, and import into Endnote X9. Then remove duplicated or ineligible studies. Screen the titles, abstracts, and full texts of all literature to identify eligible studies. All essential data will be extracted using previously created data collection sheet by 2 independent authors. Discrepancies in data collection between 2 authors will be settled down through discussion with the help of another author. The following data will be extracted from each included research: the first author's surname, publication year, language of publication, study design, sample size, number of lesions, source of the subjects, instrument, “gold standard,” and diagnostic accuracy. The true positives, true negatives, false positives, and false negatives in the fourfold (2 × 2) tables were also collected. Methodological quality was independently assessed by two researchers based on the quality assessment of studies of diagnostic accuracy studies (QUADAS) tool.^[11]^ The QUADAS criteria included 14 assessment items. Each of these items was scored as “yes” (2), “no” (0), or “unclear” (1). The QUADAS score ranged from 0 to 28, and a score ≥22 indicated good quality. Any disagreements between 2 investigators will be solved through discussion or consultation by a 3rd investigator.

### 3.2 Statistical Analysis

The STATA version 14.0 (Stata Corp, College Station, TX) and Meta-Disc version 1.4 (Universidad Complutense, Madrid, Spain) softwares were used for meta-analysis. We calculated the pooled summary statistics for sensitivity, specificity, positive and negative likelihood ratio, and diagnostic odds ratio with their 95% confidence intervals. The summary receiver operating characteristic curve and corresponding area under the curve were obtained. The threshold effect was assessed using Spearman correlation coefficients. The Cochran *Q*-statistic and *I* test were used to evaluate potential heterogeneity between studies. If significant heterogeneity was detected (*Q* test *P* < .05 or *I* test > 50%), a random effects model or fixed effects model was used. We also performed sub group and meta-regression analyses to investigate potential sources of heterogeneity. To evaluate the influence of single studies on the overall estimate, a sensitivity analysis was performed. We conducted Begg funnel plots and Egger's linear regression tests to investigate publication bias.

### 3.3 Ethics and dissemination

We will not obtain ethic documents because this study will be conducted based on the data of published literature. We expect to publish this study on a peer-reviewed journal.

## 4 Discussion

Ultrasound guided prostate puncture is the most important way for clinical diagnosis of prostate cancer.^[11]^ With extended biopsy, the positive rate was not significantly increased, but the puncture complications were significantly increased.^[12]^ To improve the accuracy rate of prostate cancer puncture and reduce the complications and pain of patients is the focus of prostate puncture research. Studies suggested that SMI is helpful to improve the accuracy of prostate cancer puncture. However, no study has published focusing on the systematic and comprehensive summary of the existing clinical evidence, which may restrict its application. In this study, we will perform a systematic review to summarize high-quality studies and to provide evidence on the evidence-based medical support for clinical practice.

## Author contributions

**Conceptualization:** Ye Tao.

**Data curation:** Cong Wang.

**Methodology:** Ye tao.

**Writing – original draft:** Cong Wang.

**Writing – review & editing:** Cong Wang and Ye Tao.
